# Treatment strategy on traumatic mid-lumbar spondyloptosis with concomitant multiple injuries: A case report and literature review

**DOI:** 10.1016/j.cjtee.2022.06.006

**Published:** 2022-06-28

**Authors:** Lin Cheng, Cheng Qiu, Xin-Yu Liu, Xi-Guang Sang

**Affiliations:** a Department of Emergency Medicine, Qilu Hospital of Shandong University, Jinan, 250012, China; b Department of Orthopaedic Surgery, Qilu Hospital of Shandong University, Jinan, 250012, China; c Cheeloo College of Medicine, Shandong University, Jinan, 250012, China

**Keywords:** Spondyloptosis, Multiple trauma, Fracture dislocation, American Spinal Injury Association, Lumbar spine

## Abstract

Spondyloptosis in the clinic is rarely reported. We herein present a 47-year-old female, who suffered from a crush injury directly by a heavy cylindrical object from the lateral side. She was diagnosed to have traumatic L_3_ spondyloptosis with multiple traumas. Staged surgical procedures were conducted and a three-year follow-up was obtained. Eventually, normal spinal alignment was restored, and neurological deficits were gradually improved. At three years follow-up, the motor strength scores and function of the sphincters were incompletely improved. Previously published reports on traumatic lumbar spondyloptosis were reviewed and several critical points for management of this severe type of spinal injury were proposed. First, thoracolumbar and lumbosacral junction were mostly predilection sites. Second, numerous patients involving traumatic lumbar spondyloptosis were achieved to American Spinal Injury Association grade A. Third, lumbar spondyloptosis was commonly coupling with *cauda equina* injury. Finally, the outcomes were still with poorly prognosis and recovery of patients was correlation to spondyloptosis severity. Based on this case report and literatures review, we highlighted that the spinal alignment restoration relying on staged operations and following rehabilitation hereof are both important once facing with multiple traumas. Furthermore, we suggested to perform routine CT angiography during lumbar spondyloptosis to justify whether there are large vessel compression or injury.

## Introduction

According to Meyerding classification, spondyloptosis is described as a unique Grade V spondylolisthesis, which presents as beyond 100% subluxation and dislocation during two vertebrae.^1^ Although spondyloptosis cases are rarely reported in the clinic, this injury still needs to be stressed in view of the poor prognosis and outcomes due to the complete loss of spinal column alignment.^2^

The characteristics of spondyloptosis mainly include congenital dysplasia of the spinal column, high-energy trauma, infective disorders, ever-evolving degeneration, and neurofibromatosis.^3^, ^4^, ^5^ For traumatic spondyloptosis, traffic accidents and fall from height are major causing factors.^6^ Traumatic spondyloptosis is rigidly associated with neurological deficits, resulting in paraplegia in about 80% of cases.^7^ Herein, we report a rare severe case of traumatic mid-lumbar spondyloptosis with multiple traumas. Staged surgical operations were performed. At three years follow-up, the patient's neurological function was partially improved. This report also reviewed related literatures on traumatic lumbar spondyloptosis and proposed several specific points on the management of mid-lumbar traumatic spondyloptosis.

## Case report

**Fig. 1:**
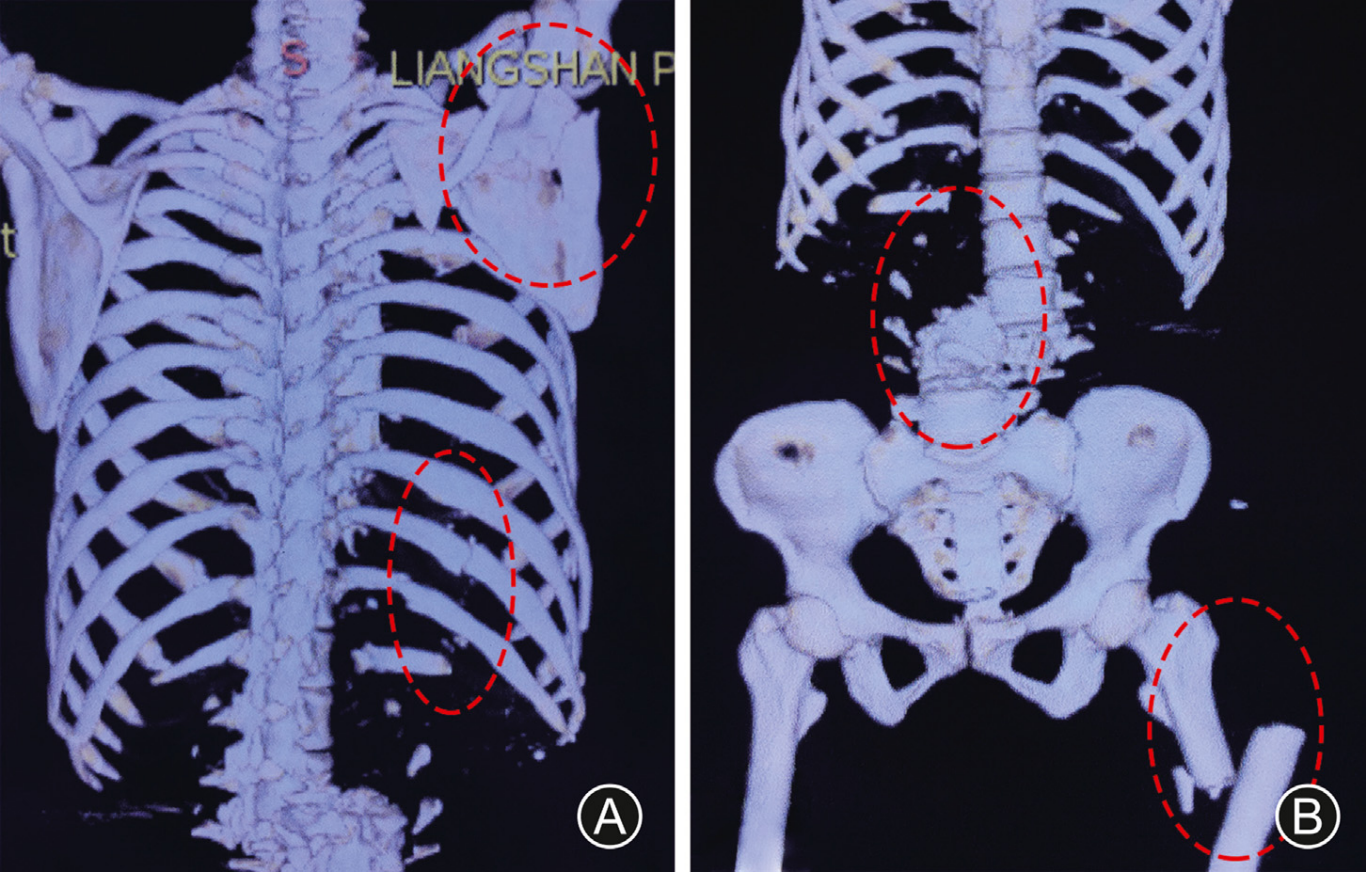
Three-dimensional reconstruction of CT scans in local hospital. Scapular fractures and rib fractures (A), L_3_ spondyloptosis combined with proximal femoral fracture and lumbar vertebra fracture (B) were enclosed by the red ellipse.

A 47-year-old female sustained a crush injury by a heavy cylindrical object from her right side. The patient complained of severe lower back pain, right shoulder pain, and thoracalgia. Radiological examinations in local hospital revealed multiple injuries, comprising L_3_ vertebra fracture-dislocation, scapular fracture, rib fractures, and left femoral shaft fracture (Fig. 1).

**Fig. 2:**
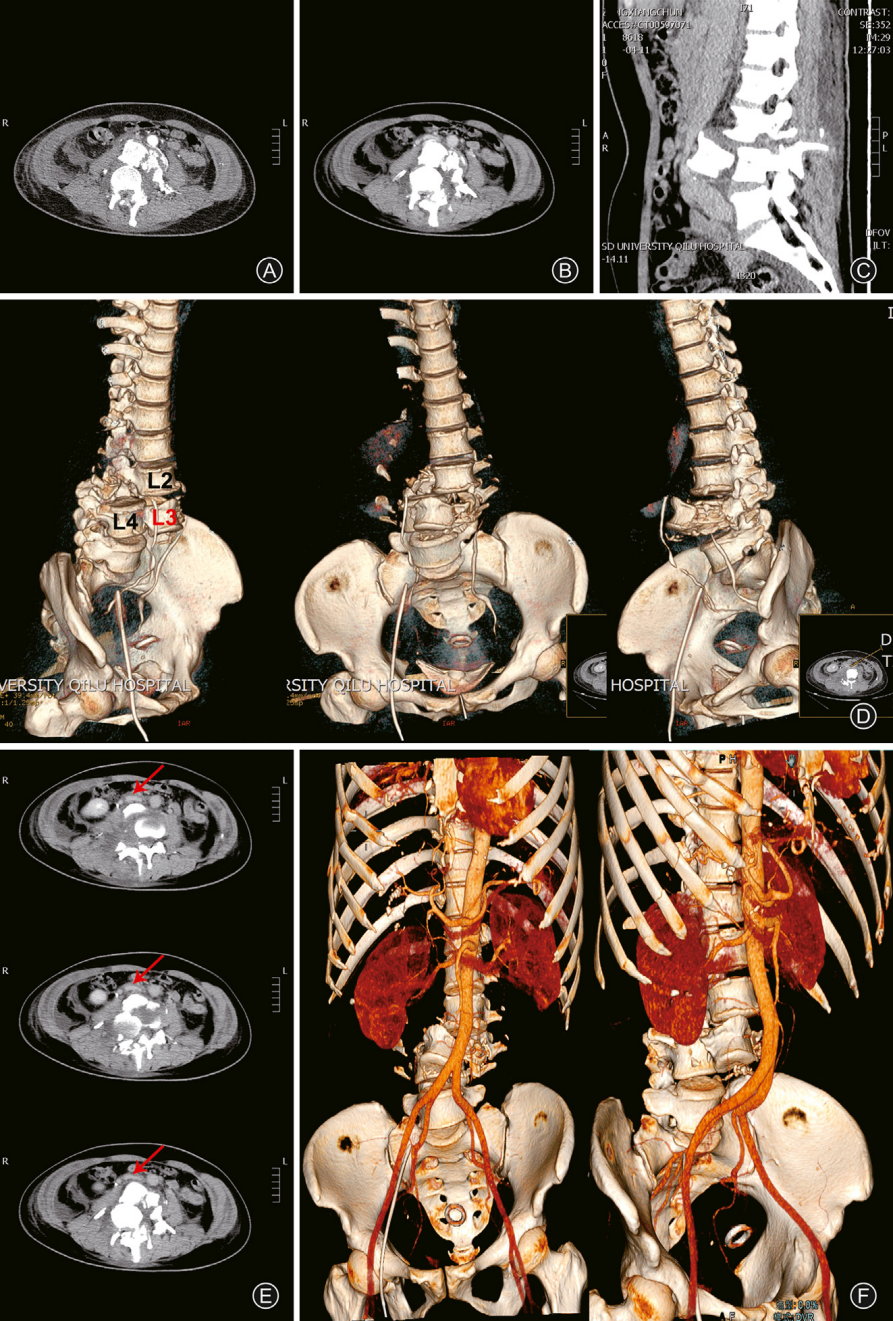
Detailed L_3_ spondyloptosis and its relationship with anterior large vessels. Complete anterior spondyloptosis of L_3_ was confirmed by two separate intensified or non-intensified CT planes (A and B), sagittal scan (C) and reconstruction images (D). Further CT angiography was conducted, compression of large vessels comprising both inferior vena cava and abdominal aorta were discovered from several CT axial planes (E) and reconstruction images (F).

The patient was managed following the advanced trauma life support protocol in the local hospital and then was transferred to our hospital for further treatment. Making an accurate diagnosis commonly depends on the stability of vital signs and a comprehensive evaluation rely on damage control. On admission, the patient was conscious and hemodynamically stable, classified as American spinal injury association (ASIA) grade A. In this patient, injury severity score (ISS) and abbreviated injury score (AIS) reached 43 and 15 respectively so that regarded as severe injury. Physical examination found shortened, swollen, and malformed left lower extremity. There exists paraparesis, including limited activity in the bilateral lower extremity and dysfunctional urination and defecation. Perirectal sensation diminished and anal sphincter tone was absent. Spinal CT indicated a complete anterior spondyloptosis of L_3_ to inferior vertebral body, and concomitant fractures. The L_3_ vertebra was downright parallel to L_4_ vertebra (Figs. 2A–C). L_3_ vertebral fracture was confirmed by three-dimensional reconstruction (Fig. 2D). But the anterior dislocation of lumbar vertebra associated with vertebral body fracture and the position with large vessels required to be further clarified. As a result, compression of both inferior vena cava and abdominal aorta was implied on consecutive CT axial planes and CT angiography (Figs. 2E and F). Blood test showed an elevated level of D-dimer.

**Fig. 3:**
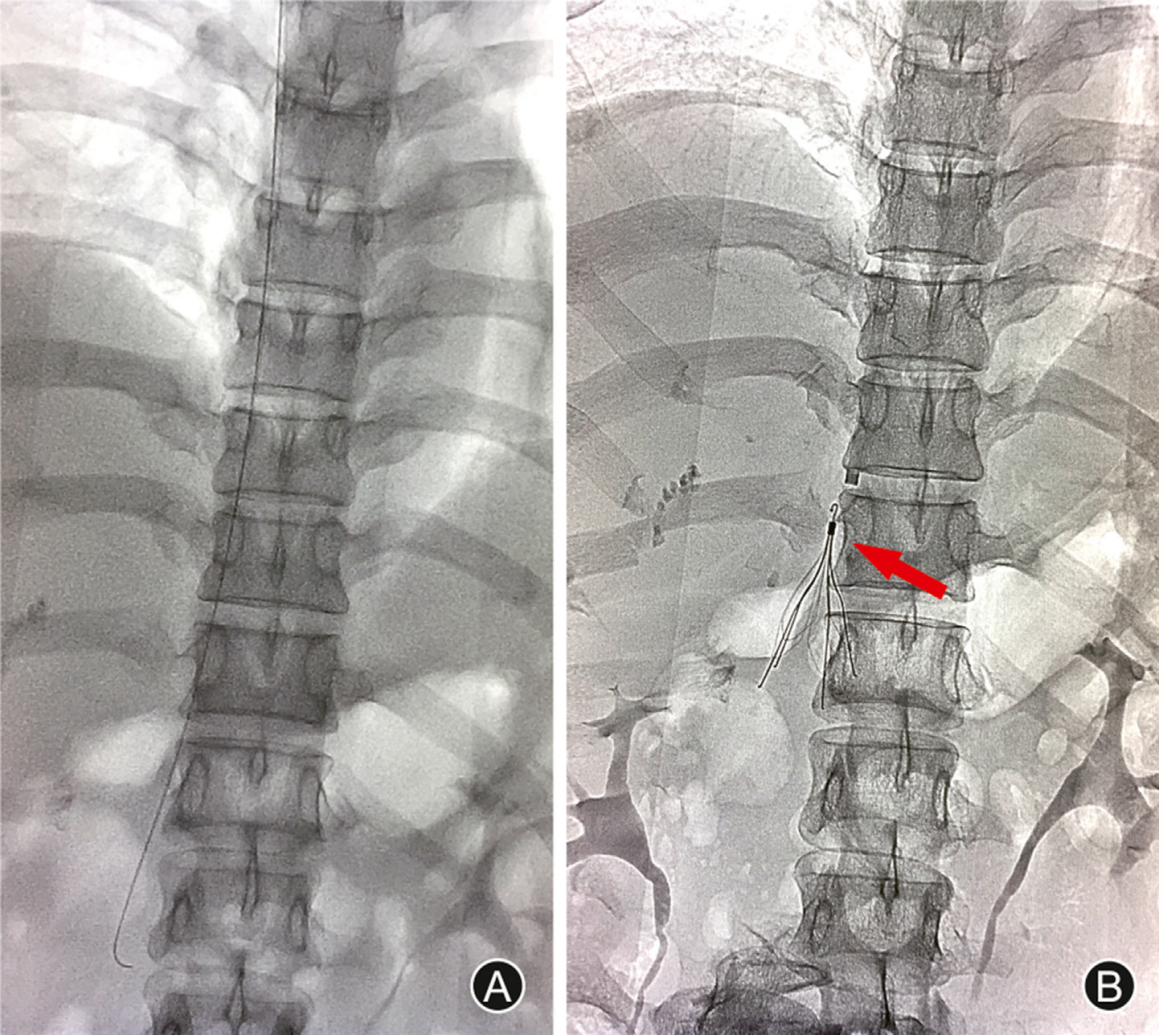
The placement of vena cava filter. To prevent thrombosis caused by vascular stenosis, a vena cava filter was imbedded into the inferior vena cava and the position was ensured (A and B).

**Fig. 4:**
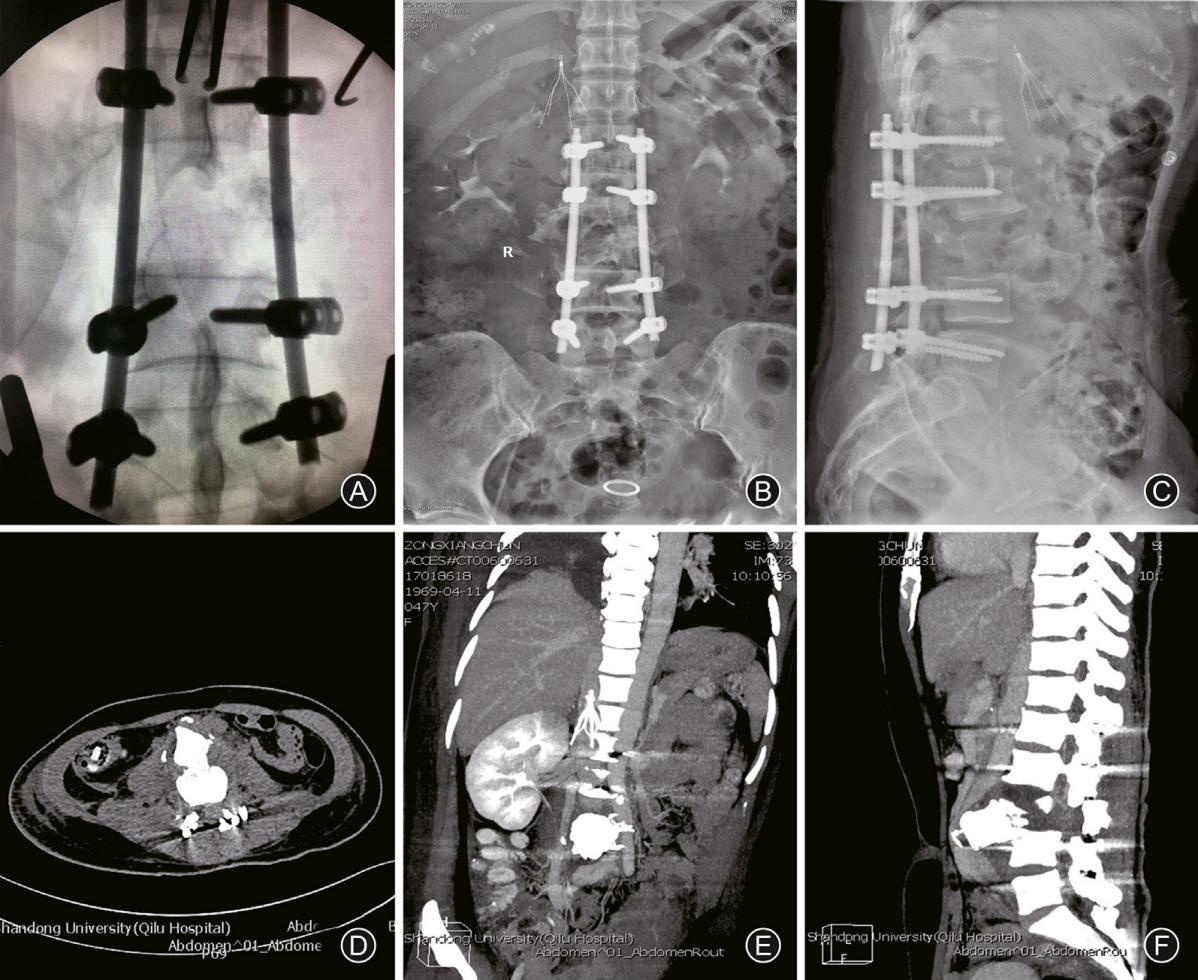
First posterior internal fixation. To stabilize the spinal alignment balance, posterior bilateral pedicle screw fixation and rods construction were performed on segment L_1_, L_2_, L_4_, and L_5_ vertebral bodies. Intraoperative fluoroscopy (A) and postoperative radiography, coronal (B) and sagittal (C), demonstrated successful restoration. Cut plane (D), coronal (E) and sagittal (F) CT scan revealed a mild retraction of L_3_ vertebra.

The strategy of staged surgical procedures was drafted in concept of orthopedic damage control. The L_3_ spondyloptosis was closely adjacent to the inferior vena cava and abdominal aorta, leading to thrombosis of the left common iliac vein. The risk of perioperative incidence of pulmonary embolism (PE) was significantly increased. Venography demonstrated filling defect at the origin of the inferior vena cava and the lumen stenosis was about 90% (Supplementary video). Therefore, inferior vena cava filter was implanted through the internal jugular vein (Figs. 3A and B). To stabilize the spinal alignment, posterior bilateral pedicle screw fixation and rods construction were performed on segments L_1_, L_2_, L_4_, and L_5_ vertebral bodies in prone position (Figs. 4A–C). Intraoperatively it was found that the *cauda equina* was injured and right nerve root of L_3_ was lacerated. A tiny tear of the dura mater was noted, which was covered with gelatin sponge. Layer by layer watertight sutures were ensured. Fortunately, there was no postoperative cerebral spinal fluid leakage. Satisfactory spinal sagittal balance was restored.

Postoperative CT and three-dimensional reconstruction of CT angiography demonstrated that the main body of the fractured L_3_ vertebrae was retracted a little while at the expense of graver compression of the abdominal large vessels (Figs. 4D–F).

**Fig. 5:**
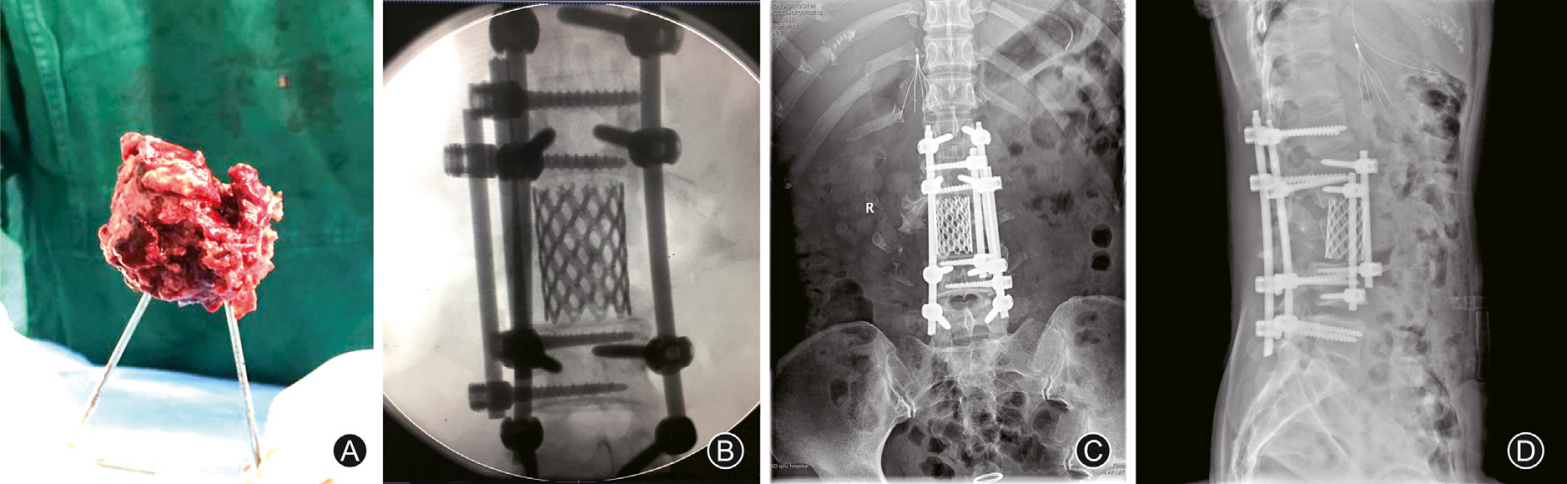
Second anterior internal fixation. We performed vertectomy of the fractured L_3_ vertebrae body (A) and placed a cage between L_2_ and L_4_ (B). Morselized bone harvested from L_3_ was packed into cage. The spinal alignment was restored as implied by postoperative radiography plain of coronal (C) and sagittal (D).

A II stage surgical intervention was performed on postoperative day 5. After anesthesia, the patient was placed in right lateral prone position, and the femoral shaft fracture was fixed by anterograde intramedullary interlocking nail. Further anterior spinal exposure was carried out through left anteriorlateral approach. Intraoperatively, it was observed that the abdominal aorta was elevated by the dislocated vertebrae body of L_3_. Then the completely fractured L_3_ vertebrae body was removed en bloc (Fig. 5A) and a cage of 26 mm in diameter with cancellous bone from L_3_ vertebrae was implanted between L_2_ and L_4_ (Fig. 5B). Reconstruction of the anterior sagittal spinal balance was enhanced by pedicle screw system, which has been implanted into L_2_ and L_4_. Postoperative radiography (Figs. 5C and D) demonstrated a restored stability of the spine and decompression. The patient was discharged and referred for further rehabilitation in a local hospital.

**Fig. 6:**
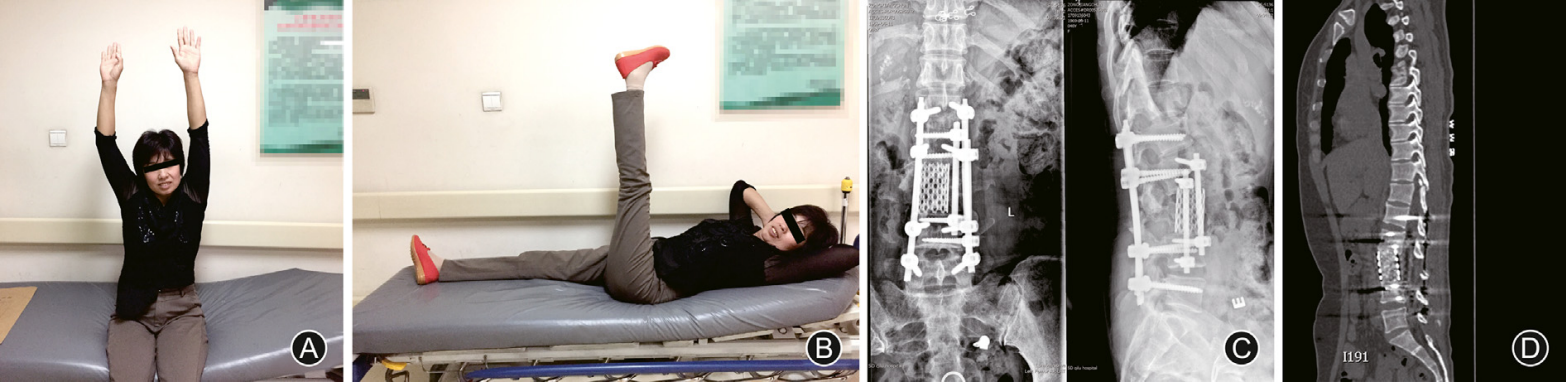
Rehabilitation functions at postoperative 6-month follow-up. At postoperative 6-month, this patient was identified to have incomplete neurological improvement (A and B). Postoperative radiography plain (C) and CT scan (D) demonstrated a stable spinal alignment.

Scheduled postoperative course was obtained. The patient returned for removal of the inferior vena cava filter at 1 month after the primary surgery. At 6-month follow-up, the patient's neural compromise had a gradual improvement. The patient had motor strength scores of 4/5 in left hip flexors and knee extensors, and 3/5 in foot dorsiflexion and plantar flexion, while 2/5 in right lower extremities (Figs. 6A and B). However, perirectal sensation was still diminished accompanied with overflow incontinence and relaxation of the anal sphincter tone. Sensation at the right lower limb (right medial and left lateral zone) disappeared. Plain radiography (Fig. 6C) and CT sagittal reconstruction (Fig. 6D) indicated a stabilized spinal alignment balance.

## Discussion

In this report, we described a rare severe traumatic mid-lumbar spondyloptosis case with multisystem damages. The shear-type mechanism of injury was caused by lateral crushing from the right side. The fractured L_3_ vertebral body was dislocated in front of L_4_ vertebra, meanwhile, the L_2_ vertebral body was displaced laterally with respect to the L_3_ vertebra body. In this rare situation, there were two types of spondyloptosises including L_2/3_ in coronal plane and L_3/4_ in sagittal plane, which had not been reported in the literatures before. The fractured L_3_ vertebral body was a free bone fragment without any connection to adjacent vertebraes. In this special situation, vertebral displacement cannot be completely corrected through single posterior surgical approach. The staged surgical strategy combined with anterior-posterior pedicle screw systems was the definitive fixation plan for this challenging traumatic spondyloptosis cases. Segmental of spinal fusion is determined by the severity of the traumatic spondyloptosis. In this case, posteriorlateral fusion in L_1-5_ levels and anterior fusion in L_2-4_ levels were applied respectively. Stabled segmental fixation systems restored the spinal sagittal and coronal balance to facilitate rehabilitation.

According to the available literatures, a total of 40 traumatic lumbar spondyloptosis cases (from year 1999–2020) have been reported.^1^^,^^2^^,^^6^^,^^8^, ^9^, ^10^, ^11^, ^12^, ^13^, ^14^, ^15^, ^16^, ^17^, ^18^, ^19^, ^20^, ^21^, ^22^, ^23^, ^24^, ^25^, ^26^, ^27^, ^28^, ^29^ Their demographics and clinical characteristics are shown in Table 1. Herein, the age range of patients reported previously was 3–56 years. Among them, males contributed the majority, 82.5%. Motor vehicle accident (25%), fall from height (25%), and crushing accident (22.5%) were the main causes. The injury mechanism is attributed to severe shear force and hyperextension. Moreover, thoracolumbar junction (25%) and lumbosacral junction (27.5%) were mostly predilection sites which may be closely association with structural development. About 35% patients with traumatic lumbar spondylopsis had concomitant severe injuries. In addition, about 75% patients involved in traumatic lumbar spondyloptosis achieved ASIA grade A and only a few of them escaped the disaster of paraplegia of the lower extremity. Furthermore, lumbar dislocation-fracture was commonly coupled with *cauda equina* injury, leading to micturition and defecation problems. Collectively, surgical interventions, involving anterior, posterior, or combined anterior-posterior pedicle screw systems, are the most common approaches for spondyloptosis management. Finally, the prognosis was poor and the recovery of patients was correlated with the spondyloptosis severity.

**Table 1:** Previous reported traumatic lumbar spondyloptosis.

Year	Authors	Age (year)	Gender	Mechanism of Injury	Affected level	Other severe injuries	ASIA grade	Neurological deficits	Surgical treatment	Outcomes
1999	Kaplan et al.^8^	18	M	MVA	L_5_-S_1_	None	NA	Yes	First L_4_-L_5_ and L_5_-S_1_ discectomy, L_5_ and S_1_ laminectomy, posterior PSF at L_3_, L_4_, and S_1_, arthrodesis from L_4_-S_1_; Second tibial allograft, anterior fixation of L4 and S_1_	Ambulation recovered
2003	Meneghini and DeWald^9^	15	F	MVA	L_5_-S_1_	None	A	Yes	Posterior PSF of L_4_, L_5_, and S_1_, intrasacral fixation by Cotrel-Dubousset instrumentation	Partial neurological improvement
2007	Bellew and Bartholomew^2^	36	F	MVA	L_2_-L_3_	Open knee joint	A	Yes	Laminectomy of L_2_, posterior PSF of T_11_-L_1_ and T_3_-S_1_, autograft	Complete neurological improvement
2008	Yadla et al.^10^	21	M	MVA	L_1_-L_2_	Pneumothorax, renal and splenic laceration and scapular fracture	C	Yes	Early splenectomy, posterior T_10_–L_4_ fusion, L_1_-L_3_ laminectomy, partial L_1_-L_2_ corpectomy	Ambulation recovered
		44	M	Roof collapse	T_12_-L_1_	Significant scalp laceration	A	Yes	Posterior T_10_–L_3_ fusion, anterior T_11_-L_2_ fusion, partial L_1_ corpectomy	No neurological improvement
2009	Daniels et al.^11^	38	M	MVA	L_4_-S_1_	None	E	No	First laminae, pedicles, and facet joints of L_4_-L_5_ were excised, posterior PSF of L_2_-S_1_, L_2_-L_3_ posterior arthrodesis; Second anterior diskectomy and fusion of L_3_–L_4_, L_4_–L_5_, and L_5_–S_1_	Unchanged
2009	Cherian and Dhawan^12^	4	F	FFH	L_1_-L_2_	None	A	Yes	L_2_ and L_3_ laminectomy, laminar hooks and rods fixation	Left limb neurological improvement
2009	Verhelst et al.^13^	6	M	CA	L_5_-S_1_	Morel-Lavalle´e lesion over the left hip and gluteal area	A	Yes	L_4_-S_1_ laminectomy, posterior PSF of L_3_, L_4_, S_1_, and S_2_	No neurological improvement
2010	Zhou et al.^14^	19	M	CA	L_4_-L_5_	None	A	Yes	Laminectomy, foraminotomy, posterior PSF of L_4_-L_5_, interbody fusion between L_4_ and L_5_, bilateral posterolateral arthrodesis by autograft from posterior iliac crest and the laminectomy	Complete neurological improvement
		13	M	RTA	L_4_-L_6_	None	A	Yes	Posterior laminectomy of L_5_ vertebral body, PSF of L_3_, L_4_, and S_1_, bilateral posterolateral arthrodesis by autograft from posterior iliac crest and the laminectomy of L_5_ vertebral body	Complete neurological improvement nearly but with right drop-foot
2011	Chandrashekhara et al.^1^	10	M	CA	L_4_-L_5_	None	A	Yes	L_2_, L_3_, L_4_, and L_5_ PSF and rod connection	Mild improvement
		16	M	MVA	L_3_-L_4_	None	A	Yes	PSF at L_1_-L_2_ and L_4_-L_5_	No improvement
		20	M	FFH	T_12_-L_1_	None	A	Yes	PSF at T_11_ and L_2_	No improvement
2011	Wilkinson et al.^15^	16	F	MVA	L_3_-L_4_	Right ulnar fracture and ischial avulsion fracture, pneumothorax, right adrenal hemorrhage, hepatic contusion, and retroperitoneal hematoma	A	Yes	PSF of L_1_-L_3_, L_5_-S_1_, and the iliac crests, laminectomy from L_3_ to L_5_, distractible cage placed with local autograft from the vertebrectomy of L_4_	Incomplete neurological improvement
2012	Wangtaphan et al.^16^	45	M	Roof collapse	L_5_-S_1_	None	A	Yes	Lumbar and sacral in situ stabilization by L_3_ and S_1_ PSF, L_5_-S_1_ fusion by L_5_ vertebral autograft	Complete neurological improvement
2013	Goni et al.^17^	32	M	CA	L_5_-S_1_	None	E	Yes	None	Free from low back pain
2013	Francis et al.^18^	19	M	MVA	L_3_-L_4_	Bilateral pulmonary contusions	B	Yes	PSF of L_1_-L_2_ and L_4_-L_5_, autograft	NA
2014	Akesen et al.^19^	28	M	CA	L_5_-S_1_	None	E	No	First laminectomy of L_5_ and inferior and superior parts of the laminae of L_4_ and S_1_, posterior instrumentation from L_4_-S_1_; Second anterior lumbar interbody fusion	Fecal and urinary incontinence improvement
2014	Amesiya et al.^20^	38	M	Concrete wall collapse	L_4_-L_5_	None	A	Yes	L_4_ laminectomy, PSF of L_4_-L_5_	Partial neurological improvement
2015	Baek et al.^21^	57	M	CA	L_5_-S_1_	Crescent fracture of the pelvis	A	Yes	Posterior bilateral L_4_, L_5_, and S_1_ PSF and iliac screws fixation	Incomplete neurological improvement
2015	Mishra et al.^6^	45	M	RTA	T_12_-L_1_	Retroperitoneal hematoma	A	Yes	L_2_-L_3_ laminectomy	No improvement
		25	M	FFH	L_1_-L_2_	None	A	Yes	L_1_-L_2_ laminectomy, T_11_-T_12_ and L_1_-L_2_ PSF	No improvement
		20	M	FFH	T_12_-L_1_	None	A	Yes	T_12_-L_1_ laminectomy, T_10_-T_11_ and L_1_-L_2_ PSF	No improvement, leg amputation due to bed sore
		18	M	FFH	L_1_-L_2_	T9 compression fracture	A	Yes	L_2_ corpectomy, T_12_-L_1_ and L_3_-L_4_ PSF	Death
		35	M	FFH	T_12_-L_1_	None	A	Yes	T_11_-L_3_ laminectomy, L_1_ partial corpectomy, T_11_-T_12_ to L_2_-L_3_ PSF	No improvement
		12	M	CA	L_4_-L_5_	None	A	Yes	L_2_-L_5_ PSF	No improvement, development of lumbar hernia
		30	M	RTA	T_12_-L_1_	Right-side pneumothorax	A	Yes	T_12_, L_1_, and L_3_-L_4_ PSF	No improvement
		40	M	FFH	L_1_-L_2_	None	A	Yes	T_11_-T_12_ and L_1_-L_2_ PSF	No improvement
		25	M	RTA	T_12_-L_1_	Head injury	A	Yes	Partial L_1_ corpectomy, L_1_ laminectomy, T_11_-L_2_ PSF	Death
		35	M	CA	L_1_-L_2_	None	A	Yes	T_11_-T_12_ and L_3_-L_4_ PSF	Death
		22	F	FFH	T_12_-L_1_	None	A	Yes	T_11_-T_12_ and L_1_-L_2_ PSF	No improvement
2015	Sandquist et al.^22^	20	M	MVA	T_12_-L_1_	None	A	Yes	L1 vertebrectomy, PSF of T_8_-L_4_, T_12_-L_2_ interspace autograft from L1 vertebrectomy	No neurological improvement
2015	Gabel et al.^23^	27	M	MVA	L_5_-S_1_	Massive trauma to the right lower quadrant of the abdomen	A	Yes	L_2_ to sacroiliac posterior instrumented fusion with L_5_ vertebrectomy and placement of an interbody cage	No neurological improvement
2017	Tandon et al.^24^	3	F	Fall on the ground	L_5_-S_1_	None	E	No	Posterior L_3_-S_2_ PSF	Pain-free
2018	Yamaki et al.^25^	4	F	Wall collapse	L_5_-S_1_	None	D	No	L_5_-S_1_ discectomy, anterior plaque fixation of L_5_-S_1_, L_4_-L_5_ laminectomy, PSF at L_2_-L_3_ and a crest iliac screw fixation	No improvement
2018	Garg et al.^26^	18	M	RTA	L_1_-L_2_	None	A	Yes	T_12_-L_1_ and L_3_-L_4_ PSF, L_2_ corpectomy	No improvement
		50	M	RTA	T_12_-L_1_	None	A	Yes	T_10_-T_12_ and L_2_-L_3_ PSF, L_1_ corpectomy	No improvement
2019	Cabrera et al.^27^	42	M	CA	L_3_-L_4_	Ribs fracture, left hemopneumothorax, retroperitoneal hematoma	NA	Yes	L_3_ vertebrectomy, Installation of an expandable cage between L_2_ and L_4_, posterior and anterior PSF at L_2_ and L_4_	Partial neurological improvement
2020	Xu et al.^28^	42	M	FFH	L_3_-L_4_	None	E	No	L_3_-L_4_ fusion, PSF at L_2_-L_4_ and S_1_	Pain-free
2020	Zhao and Lan^29^	56	M	FFH	L_2_-L_3_	Ribs fracture, right hemopneumothorax	A	Yes	L_2_ corpectomy, L_2_ vertebra body in situ autograft, T_12_ to L_4_ posterior fusion	Partial neurological improvement

ASIA: American spinal injury association M: male; F: female; MVA: motor vehicle accident; FFH: fall from height; CA: crushing accident; RTA: road traffic accident; NA: not available; PSF: pedicle screw fixation.

Spondyloptosis leads to severe spinal mal-alignment, sagittal or coronal planes dislocation.^2^^,^^14^ The occurrence position of spondyloptosis could be unequivocally on cervical, thoracic, lumbar, or even sacral spine through radiological examinations, resulting in serous neurological deficits.^18^^,^^30^, ^31^, ^32^ Current surgical interventions are commonly conducted on reduction of spine column as well as decompression and stabilization of associated dislocation-fracture segments. Notably, the following rehabilitation trainings are important to the grade of neurological improvements, whereas, the recovery outcomes tend to be patient specific. Based on previously published studies, conservative treatment is not recommended for spondyloptosis irrespective of symptoms status. A persistent unstable spinal alignment eventually leads to spinal deformity (kyphosis or lordosis), which might be associated with low back pain.^16^^,^^33^ Meanwhile, early surgical reduction may be beneficial by improving neurological symptoms.

Spondyloptosis located on mid-lumbar spine is sparse in the clinic. Considering that the anatomic situations of large vessels such as abdominal aorta and inferior vena cava are closely contiguous to lumbar vertebrae.^18^ The major issue is to imperil large vessels in abdomen on extrusion and even rupture threat. Overall anterior, posterior or lateral dislocation of superior vertebral body for inferior vertebra exacerbate volume jostling at the same segmental axial plane. Lumen stenosis and congestion of large vessels is advantageous to thrombosis formation which is causative for pulmonary embolism attack that serves as the most troublesome for survival. Moreover, bony fragments from vertebra fractures are easily embed in these vessels that lead to unbearable results. Therefore, routine CT angiography is suggested to evaluate whether large vessel injury exist in terms of the considerations above.

Traumatic mid-lumbar spondyloptosis with polytrauma is a challenging situation to manage in the clinic. Herein, we presented a case of a complete L_3_ spondyloptosis combined with polytrauma through the whole body. The current report mainly focused on spondyloptosis and combined injuries according to the concept of damage control orthopaedics. The spinal alignment restoration relying on individual staged operations and subsequent rehabilitation are the key points. For patients with multiple injuries, especially those with spinal fractures and dislocations, it is necessary to pay attention to the first choice of early appropriate spinal intervention and to evaluate the degree of pressure near the vessels by computed tomography angiography before operation. These patients are mostly at a high risk for deep vein thrombosis and pulmonary embolism. It is recommended to implant intravenous vena cava filter through internal jugular vein. Meanwhile, for patients with better functional recovery, an observation of the posterolateral spinal fusion in the follow-up process is essential to avoid failure of spinal implant fixation.

Taken together, based on the major premise of stable vital signs, strategy of staged surgical procedures for spondyloptosis is dominate in terms of orthopedic damage control. Early spinal alignment reestablishment is commonly regarded as the superior choice relative to the fixation of limb fractures in spondyloptosis which accompanied with concomitant multiple injuries. Ultimately, the imperative of postoperative rehabilitation exercise is also critical for the functional recovery.

## Funding

Nil.

## Ethical statement

This study was approved by the Institution Review Board of Qilu Hospital of Shandong University and written informed consent was obtained from this patient.

## Declaration of competing interest

The authors declare that they have no competing interests.

## Author contributions

Lin Cheng and Cheng Qiu conducted literature research,collected data, and drafted and revised the manuscript. Xin-Yu Liu and Xi-Guang Sang revised the manuscript. All authors read and approved the final version of manuscript.
